# Supplementary material for: D-Dimers Level as a Possible Marker of Extravascular Fibrinolysis in COVID-19 Patients
Source: J Clin Med. 2020 Dec 24;10(1):39. doi: 10.3390/jcm10010039 (PMC7795726; doi:10.3390/jcm10010039)
Supplement: Supplementary file 1 [file jcm-10-00039-s001.pdf]

Supplementary Material

Table S1. Lung parenchymal involvement assessed by chest computed tomography according to D-dimers staging at peak.

	**D-Dimers Staging at Peak**			***p* value**
	**Stage 1 <3 ULN (*n* = 67)**	**Stage 2 3–6 ULN (*n* = 30)**	**Stage 3 >6 ULN (*n* = 26)**	
Minimal (<10%)	10 (14.9)	2 (6.7)	1 (3.8)	0.001
Moderate (10–25%)	27 (40.3)	12 (40.0)	4 (15.4)	
Severe (25–50%)	22 (32.8)	10 (33.3)	6 (23.1)	
Critical (>50%)	8 (11.9)	6 (20.0)	15 (57.7)	

Data are presented as *n* (%). Abbreviation: ULN, upper limit of normal.

Table S2. Outcomes according to D-Dimers staging at admission.

	**D-Dimers Staging at Admission**			***p* value**
	**Stage 1 <3 ULN (*n* = 79)**	**Stage 2 3–6 ULN (*n* = 23)**	**Stage 3 >6 ULN (*n* = 16)**	
Critical pulmonary injuries (>50%)	11 (13.9)	9 (39.1)	7 (43.8)	0.004
Transfer to ICU or in-hospital death	22 (27.8)	12 (52.2)	5 (31.2)	0.091
ICU Transfer	22 (27.8)	11 (47.8)	5 (31.2)	0.196
In-hospital death	0 (0.0)	1 (4.3)	1 (6.2)	0.115
Discharged alive	65 (95.6)	20 (90.9)	13 (86.7)	0.400
Lung injuries extent Minimal (<10%)	12 (15.2)	1 (4.3)	1 (6.2)	0.038
Moderate (10–25%)	32 (40.5)	5 (21.7)	4 (25)	
Severe (25–50%)	24 (30.4)	8 (34.8)	4 (25)	
Critical (>50%)	11 (13.9)	9 (39.1)	7 (43.8)	

Data are presented as *n* (%). Abbreviation: ICU, intensive care unit; ULN, upper limit of normal.

Table S3. Predictive factors for the occurrence of the composite of in-hospital death or transfer to the intensive care unit.

	Univariable Analysis		Multivariable Analysis	
	OR [95%CI]	*p* Value	OR [95%CI]	*p* Value
Age	0.98 [0.95–1.00]	0.089		
Male	2.45 [1.08–5.55]	0.031	1.49 [0.47–4.73]	0.496
BMI >30 kg/m^2	1.07 [0.47–2.43]	0.858		
Fibrinogen at admission	1.38 [0.99–1.92]	0.054		
Fibrinogen at peak	1.48 [1.12–1.95]	0.006	1.22 [0.87–1.71]	0.234
CRP at admission	1.00 [0.99–1.00]	0.128		
CRP at peak	1.01 [1.00–1.01]	<0.001	1.00 [0.99–1.01]	0.330
Leukocytes at admission	0.96 [0.85–1.09]	0.619		
Leukocytes at peak	1.00 [0.98–1.02]	0.460		
Lymphocytes at admission	0.59 [0.24–1.46]	0.258		
Procalcitonin at admission	0.93 [0.82–1.06]	0.318		
BNP at admission	0.99 [0.99–1.00]	0.301		
D-Dimers staging at admission	1.17 [0.68–1.99]	0.556		
D-Dimers staging at peak	2.91 [1.76–4.83]	<0.001	2.50 [1.27–4.93]	0.008

Abbreviations: BMI, body mass index; BNP, B-type natriuretic peptide; CI, confidence interval; CRP, C-reactive protein; OR, odds ratio.
